# Effect of self-perceived oral habits on orofacial dysfunction and oral health-related quality of life among a group of Egyptian children: a cohort study

**DOI:** 10.1007/s40368-022-00740-8

**Published:** 2022-08-24

**Authors:** M. A. A. A. Abd-Elsabour, R. M. H. Hanafy, O. M. Omar

**Affiliations:** 1 Pediatric Dentistry and Dental Public Health Department, Faculty of Dentistry, Cairo University, 11 Al Saraya street, Al Manial, Kasr Al Ainy, Cairo, Egypt; 2 Pediatric and Community Dentistry Department, Faculty of Dentistry, Ahram Canadian University, Giza, Egypt

**Keywords:** Oral habits, Children, Orofacial dysfunction, Oral health-related quality of life

## Abstract

**Purpose:**

This study aims to investigate the relationship between OHRQoL and orofacial dysfunction in children practicing oral habits.

**Methods:**

Thirty Egyptian Children, aged from five to seven years, practicing oral habits (habit practicing/exposed group), were examined for orofacial dysfunction using Nordic Orofacial Test-Screen (NOT-S). Their parents were asked to fill 8-item Parental–Caregiver Perception Questionnaire (P-CPQ), translated to Arabic, as an assessment tool for their children’s OHRQoL. The scores of the habit practicing group were compared to those obtained from another 30 children with matched criteria not practicing oral habits (habit free/ control group).

**Results:**

Children in the exposure group showed higher total NOT-S score (median 3, range 1–5) and higher P-CPQ (median 6, range 1–16) than the control group (median 0.5, range 0–2) and (median 4, range 1–8), with a statistical significance (*p* = 0.00, *p* = 0.014), respectively. A statistically significant moderate positive correlation was found between OHRQoL and orofacial dysfunction in the habit practicing group, (*R* = 0.384, *p* = 0.036). The exposure group was found to be 7.4 and 1.5 times the control group in developing orofacial dysfunction, and having inferior OHRQoL, respectively.

**Conclusion:**

An existing association between the degree of orofacial dysfunction and OHRQoL in children practicing oral habit(s) is suggested.

**Trial registration number:**

NCT04575792, date of registration: 26/9/2020, first posted (approved): 5/10/2020.

## Introduction

Quality of life (QoL) was defined by WHO as “individuals’ perception of their position in life in the context of the culture and value systems in which they live and in relation to their goals, expectations, standards, and concerns” (The Whoqol Group 1998). It was described more simply by the Centre of Health Promotion, the University of Toronto as “the degree to which an individual can enjoy the possibilities of life” (Hernández et al. 2015). This model was expanded to include almost all aspects of life, on top of which the health-related aspects. To express a health-related quality of life concept from the patient’s perspective, Health-Related Quality of Life measures, which are patient-reported outcome measures, were designed. These measures aim to interpret the effect of health condition or therapeutic measure from the patient's psychosocial point of view rather than the clinician's biomedical view (Engel 1977). Oral Health-Related Quality of Life (OHRQoL) has been investigated on widely, as an integral portion of health-related quality of life (Hernández et al. 2015). Any condition that affects the well-being of the orofacial complex will have an impact on OHRQoL. Hypodontia, amelogenesis imperfecta, early childhood caries (ECC), molar incisor hypomineralisation (MIH), cleft lip and palate, malocclusion, dental trauma, and any other oral/dental conditions are found to have its impact on OHRQoL (Berger et al. 2009; Hashem et al. 2013; Kappen et al. 2019; Montes et al. 2019; Sharna et al. 2019; Coutinho et al. 2020). Child’s OHRQoL measures are targeting the assessment of orofacial structures’ well-being and function, and their effect on the child’s emotional and social perception, from the child’s or his parents’ point of view (Thomson et al. 2013).

Orofacial Myofunctional Disorder is a group of abnormal muscular activities beyond the normal function, that results in a change in normal freeway range, a space between the dental arches at rest, and thus changes the normal posture of the tongue making it acts as a myofunctional appliance, and subsequently affects eruption of teeth. Furthermore, abnormal development of orofacial complex structure, and possible articulation and pronunciation abnormalities can occur (Mason 2005). Oral habits, as an example of orofacial myofunctional disorder*,* are defined as repeated orofacial muscular activities without a functional benefit. Many forms of oral habits could be seen in children as nail biting, finger or object sucking, lip, tongue, or cheek biting, clenching and bruxism, and mouth breathing. The exertion of minute un-opposed forces on the same area repeatedly for a long time will result in deformation of the orofacial complex, especially at the young age when the maxillofacial complex is still growing, and cause a consequent orofacial dysfunction which will, in return, affect the child's OHRQoL (Leme et al. 2013; Reyes Romagosa et al. 2014).

Sucking habits, mouth breathing, and tongue thrusting habits are found to be the most deleterious oral habits, especially in the age group of five to seven years (Kasparaviciene et al. 2014). It was found that these three deleterious habits are the most practised habits among Egyptian children ageing from six to nine years (19.6%) (Farrag and Awad 2016).

This study hypothesised a positive relationship between orofacial dysfunction and OHRQoL in children practicing oral habits in comparison to their counterparts who do not practise oral habits. Thus, this study aims to measure the orofacial dysfunction, and OHRQoL, in a group of Egyptian children aged from five to seven years, practicing oral habit(s); furthermore, to compare their results to a matching group of children who do not practise oral habit(s); and to investigate the correlation between orofacial dysfunction and OHRQoL in children practicing oral habits. Up to our knowledge, few studies investigated the effect of orofacial dysfunction on OHRQoL in children (Leme et al. 2013; Collado et al. 2017; Sardenberg et al. 2017; Montes et al. 2019), none of them was conducted on Egyptian children.

## Subjects and methods

### Study design and study variables

This study was a retrospective cohort study, in which practicing oral habit(s) was considered the exposure factor, and orofacial dysfunction and OHRQoL were considered the outcome variables.

### Study settings

The current study was conducted on Egyptian children attending the Outpatients’ Diagnostic Clinic of Pediatric Dentistry and Dental Public Health Department, Faculty of Dentistry, Cairo University, Egypt, during the period from the first of January 2021 to the first of April 2021.

### Ethical approval and trial registration

Ethical approval for the research protocol was obtained from Research Ethics Committee, Faculty of Dentistry, Cairo University, Egypt, approval number: 71120, approval date: 24/11/2020, and the trial was registered on ClinicalTrail.gov, ID: NCT04575792. This study was performed following the ethical standards as laid down in the 1964 Declaration of Helsinki.

### Sample size calculation

A power analysis was designed to have adequate power to apply a two-sided statistical test of the research question regarding the effect of practicing oral habits on orofacial dysfunction and OHRQoL among a group of Egyptian children. By adopting an alpha level of (0.05), a beta of (0.2), i.e. power = 80%, and effect size (*d*) of (0.740) calculated based on the results of Leme et al. 2013, the predicted sample size (*n*) was a total of (30) cases in each group. Sample size calculation was performed using G*Power version 3.1.9.7.

### Participants

1. *The habit practicing group (exposed group)*
  All children attending the diagnostic clinic on the days of examination reporting practicing one or more of the following habits (mouth breathing, sucking habit, bruxism, or nail biting) and fulfilling the eligibility criteria were included in this study until a total number of 30 children were recruited.
2. *The habit free group (control group)*

A similar number of children, with matched inclusion and exclusion criteria, who do not practise oral habits were included.

### Eligibility criteria

#### Inclusion criteria

- Cooperative children with an age range from five to seven years old.
- Apparently healthy children.
- Both genders.
- Children whose parents/caregivers accept to participate in this study.

#### Exclusion criteria

- Untreated caries.
- History of untreated dental trauma.
- History of orthodontic treatment
- Children having one or more of the following dental anomalies: MIH, amelogenesis imperfecta, dentinogenetic imperfecta, hypodontia, or dental fluorosis.

All the above-mentioned conditions were potential confounders, thus were treated by restriction from the study sample (Berger et al. 2009; Hashem et al. 2013; Montes et al. 2019; Sharna et al. 2019; Coutinho et al. 2020).

### Study procedures

#### Informed consent

Before starting the research, written informed consent, was signed by the parents, and a child's verbal assent were obtained after a detailed explanation of the study protocol.

### Data collection

The parents of children of both groups were asked to fill the administrative chart (age, sex, past and present medical and dental history, type of habit if present, and its duration). To minimise the potential reporting bias, the child’s parents were asked to fill the Parental–Caregiver Perception Questionnaire (P-CPQ), the Arabic version, in the waiting area, before the child was examined for orofacial dysfunction. (Thomson et al. 2013; Al-Riyami et al. 2016). After that, the child was examined by the researcher for orofacial dysfunction, using Nordic Orofacial Test-Screen (NOT-S) as an assessment tool (Bakke et al. 2007), in the Outpatients’ Diagnostic Clinic.

### Assessment tools

Nordic Orofacial Test-Screen (NOT-S) was utilised as an assessment tool for orofacial dysfunction (Bakke et al. 2007). It consists of two main parts, the interview part with six domains inquiring about the following items (sensory function, breathing, habits, chewing and swallowing, drooling, and dry mouth), while the examination part investigates (face at rest, nose breathing, facial expression, masticatory muscles and jaw function, oral motor function, and speech), Table 1. The calculated score would range from “zero” to “12”, the higher the score the worse the condition of orofacial dysfunction.

**Table 1:**
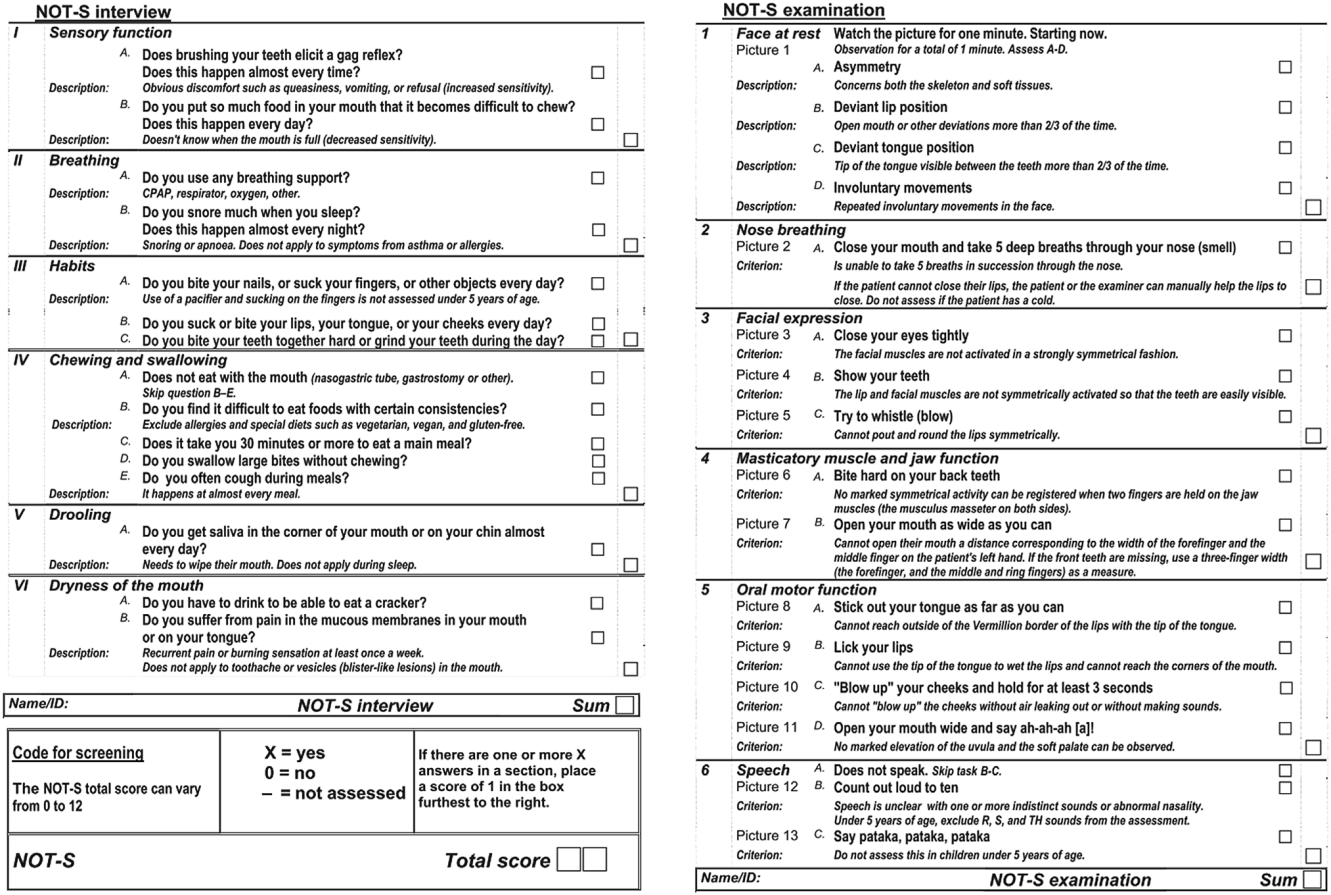
The English version of the Nordic Orofacial Test-Screen containing a structured interview (left) and a clinical examination (right), each consisting of six domains (Bakke et al. 2007)

Oral Health-Related Quality of Life (OHRQoL) was assessed using the Arabic version of the 8-item P-CPQ, validated by Al-Riyami et al. (2016). It consists of four parts (oral symptoms, functional limitation, emotional well-being, and social well-being), and was answered by the parents. The summation of the questionnaire items' scores would result in a total score ranging from “zero” to “32”, the higher the score the worse the OHRQoL (Thomson et al. 2013), Table 2.

**Table 2:** Parental–Caregiver Perception Questionnaire, 8-item short form

In the past 3 months, how often has your child had (item) because of the teeth, lips, jaws, or mouth?	
Item	Part
Pain in the teeth, lips, jaws, or mouth	Oral symptoms
Food caught in or between the teeth	Oral symptoms
Difficulty biting or chewing firm foods	Functional limitations
Taken longer than others to eat a meal	Functional limitations
Been upset	Emotional well-being
Been irritable or frustrated	Emotional well-being
Missed school or preschool	Social well-being
Not wanted to talk to other children	Social well-being

### Statistical analysis

Data management and statistical analysis were performed using the Statistical Package for Social Sciences (SPSS) version 18. Numerical data were summarised using median, range, and means. Data were explored for normality by checking the data distribution, using Kolmogorov–Smirnov and Shapiro–Wilk tests.

Most data were non-parametric and comparisons between the two groups’ NOT-S and P-CPQ scores were done using Mann–Whitney *U* test.

Correlations between different variables’ scores were performed using Spearman’s rho correlation test. The correlation coefficient was used to measure the strength of the linear association between two variables.

All *p* values are two sided. *p *values ≤ 0.05 were considered significant.

To convert the continuous variables into binary data, the scores of the two study groups were gathered, and the medians of the data of both groups gathered were used as cut-off points of the outcomes’ measures, as recommended by DeCoster et al. 2011. The NOT-S cut-off point for “developing orofacial dysfunction” was assigned at ≥ 2, which is similar to what was reported by Bakke et al. 2007, while the P-CPQ cut-off point for “having inferior OHRQoL” was assigned at ≥ 5.

Microsoft Excel 365 was utilised in generating illustrative charts.

## Results

**Fig. 1:**
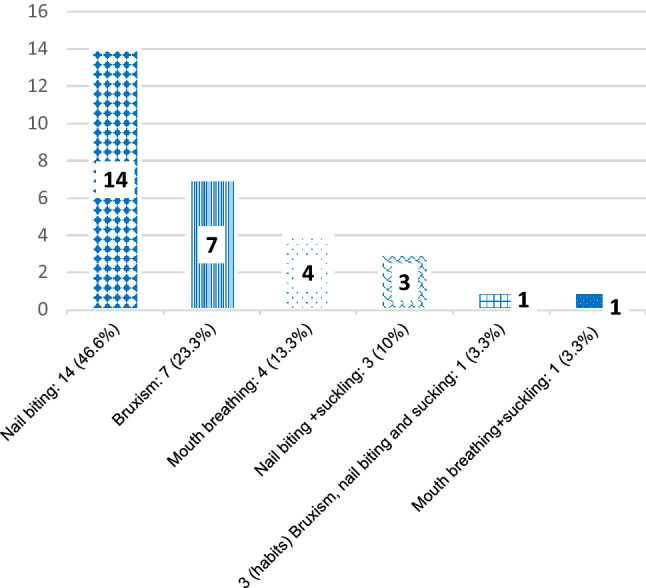
Bar chart illustrating the distribution of oral habits among the habit practicing group by number (*n*) and percentage (%)

The mean age of children was 5.78 (0.88) years in the habit practicing group, and 5.70 (0.74) years in the habit free group. The habit practicing group consisted of 13 males (43.5%) and 17 females (56.5%), while the habit free group consisted of 15 males (50%) and 15 females (50%). Nail biting was the most prevalent habit (46.7%) among the habit practicing group, followed by bruxism (23.3%) and mouth breathing (13.3%). The distribution of oral habits among the habit practicing group was demonstrated in Fig. 1.

**Fig. 2:**
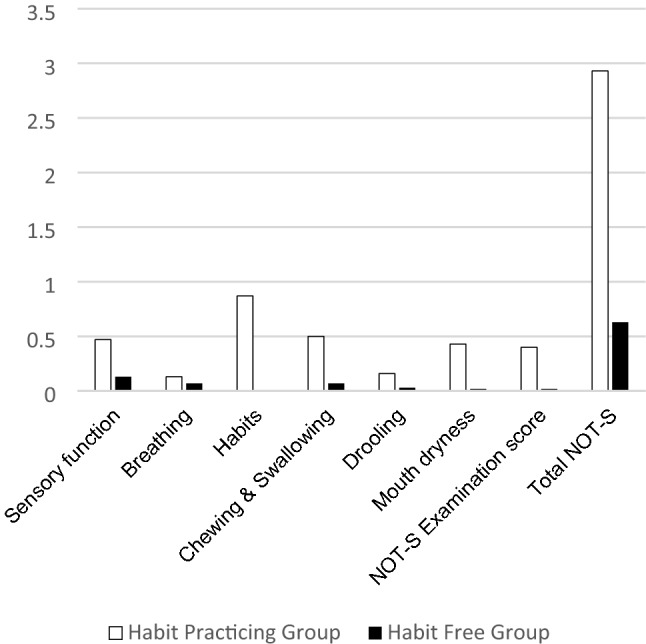
Bar chart illustrating mean scores in various Nordic Orofacial Test-Screen interview domains and examination part in habit practicing and habit free groups

Total NOT-S score in the habit practicing group recorded a median of three (range 1–5), which was significantly higher (*p* = 0.00) than the habit free group (median = 0.05, range 0–2). The habit practicing group recorded significantly higher scores for sensory function, habits, chewing and swallowing, and mouth dryness domains, (*p* = 0.005, 0.00, 0.00, and 0.025, respectively). Higher values for breathing, drooling domains, and NOT-S examination scores were also noted in the habit practicing group compared to the habit free group, with no statistically significant difference between the two groups, Table 3, and Fig. 2.

**Table 3:** Descriptive data and comparison of Nordic Orofacial Test-Screen scores in the habit practicing group and the habit free group

Study group	Habit practicing group				Habit free group				*p* value
Item	*n* (%)	Median	Range	Mean (SD)	*n* (%)	Median	Range	Mean (SD)	
Sensory function	14 (46%)	0	0–1	0.47 (0.51)	4 (13%)	0	0–1	0.13 (0.35)	0.005*
Breathing	4 (13%)	0	0–1	0.13 (0.35)	2 (6.6%)	0	0–1	0.07 (0.25)	0.39
Habits	25 (83%)	1	0–1	0.87 (0.35)	0 (0%)	0	0–1	0 (0)	0.00*
Chewing and Swallowing	14 (46%)	0	0–1	0.5 (0.5)	2 (6.6%)	0	0–1	0.07 (0.25)	0.00*
Drooling	4 (13%)	0	0–1	0.16 (0.38)	1 (3.3%)	0	0–1	0.03 (0.18)	0.088
Mouth dryness	12 (40%)	0	0–1	0.43 (0.5)	5 (16.6%)	0	0–1	0.017 (0.38)	0.025*
NOT-S Examination score	11 (36%)	0	0–1	0.4 (0.56)	5 (16.6%)	0	0–1	0.017 (0.38)	0.075
Total NOT-S	–	3	1–5	2.93 (0.94)	–	0.5	0–2	0.63 (0.72)	0.00*

*Significance level *p* ≤ 0.05, Mann–Whitney *U* test.

Regarding the NOT-S examination part, no findings were observed in 19 (63.3%) of children in the habit practicing group, in comparison to 25 (83.3%) in the habit free group. The most common detected findings were “Deviation of lip position” (incompetent lips) was detected in one patient (3.3%) in the habit free group. “Lisping at letters as (R, Th, S) or any other letter” occurred in four patients (13.3%) in both groups. “The tongue is visible between the teeth” in two cases in the habit practicing group, and it was associated with mouth breathing habit in an additional case, in addition to four children who were found to be mouth breathers in the habit practicing group.

**Fig. 3:**
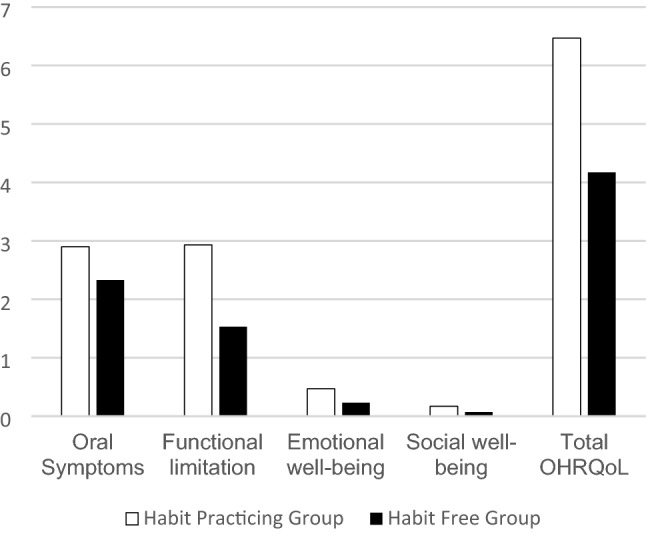
Bar chart illustrating mean values of various Parental-Caregiver Perception Questionnaire parts and total score in habit practicing and habit free groups

The total P-CPQ score in the habit practicing group recorded a median of six (range 1–16), which was significantly higher (*p* = 0.014) than the habit free group (median = 4, range 1–8). The habit practicing group recorded a significantly higher score for functional limitations, (*p* = 0.011). Higher values for oral symptoms, emotional, and social well-being were also noted in the habit practicing group, with no statistically significant difference between groups, Table 4, and Fig. 3.

**Table 4:** Descriptive data and comparison of Parental-Caregiver Perception Questionnaire scores in the habit practicing group and the habit free group

	Habit practicing group			Habit free group			*p* value
	Median	Range	Mean (SD)	Median	Range	Mean (SD)	
Oral Symptoms	2.5	0–6	2.9 (1.63)	2	0–5	2.33 (1.27)	0.218
Functional limitation	2.5	0–8	2.93 (2.2)	2	0–5	1.53 (1.46)	0.011*
Emotional well-being	0	0–3	0.47 (0.94)	0	0–3	0.23 (0.68)	0.298
Social well-being	0	0–5	0.17 (0.91)	0	0–1	0.07 (0.25)	0.584
Total OHRQoL	6	1–16	6.47 (3.79)	4	1–8	4.17 (1.91)	0.014*

*Significance level *p* ≤ 0.05, Mann–Whitney U test

**Fig. 4:**
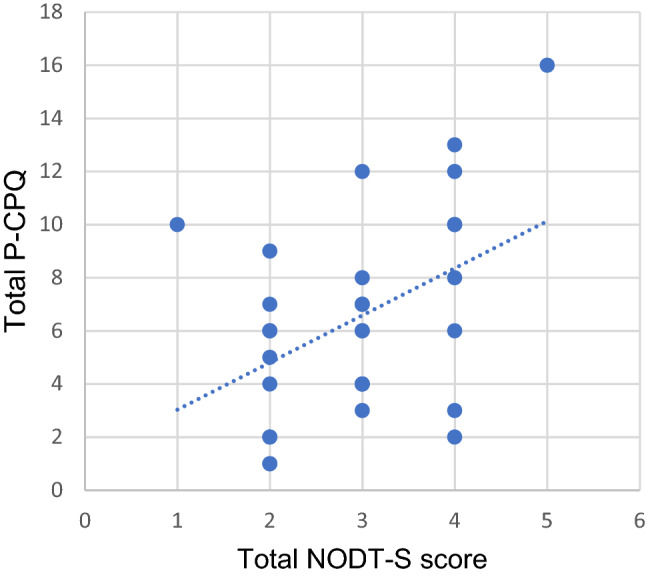
Correlation between total Nordic Orofacial Test-Screen and total Parental-Caregiver Perception Questionnaire scores in the habit practice group

Spearman’s correlation test revealed no significant correlation between age in both groups, duration of the habit in the exposed group, and different NOT-S and P-CPQ total scores and domains/parts scores. The total NOT-S score showed a statistically significant moderate positive correlation with the total P-CPQ score in the habit practicing group (*R* = 0.384, *p* = 0.036), Fig. 4. Strong positive statistically significant correlations were found between the total P-CPQ score and chewing and swallowing, and mouth dryness domains of the interview part of NOT-S, in the habit practicing group, (*R* = 0.422, *p* = 0.02, and *R* = 0.422, *p* = 0.02, respectively). While in the control group, there was no significant correlation between total NOT-S and domains, and total P-CPQ and its parts.

Calculation of the number of events per group was done based on the assumed cut-off points for each outcome measurement. The exposure group was found to be 7.4, and 1.5, times the control group in developing orofacial dysfunction, and having inferior OHRQoL respectively, Table 5.

**Table 5:** Relative risks for the study outcomes

The study outcome	Exposed group (*n* = 30)			Control group (*n* = 30)			Relative risk
	Number of events per group	Absolute risk (event rate)	Odds	Number of events per group	Absolute risk (event rate)	Odds	
Total NOT-S score	29	0.96	29	4	0.13	0.15	7.4
Total P-CPQ score	19	0.63	1.73	13	0.43	0.76	1.5

## Discussion

Practicing deleterious oral habits in children, beyond the reversible age limit, is believed to cause a harmful effect on orofacial structure, which results in orofacial dysfunction and consequently compromises OHRQoL (Leme et al. 2013).

This study was designed to be a retrospective cohort study, conducted on a group of apparently healthy Egyptian children, aged from five to seven years practicing one or more of the oral habit(s), and their scores were compared with another group of habit free children with matched criteria.

The selected age range was from five to seven years, as oral habits can be considered a normal portion of the child’s psychological development till the age of three years. (Batra et al. 2014).

Nordic Orofacial Test Screen and P-CPQ were utilised in this study due to their reliability, validity, ease of use, with clear scoring criteria that assess orofacial dysfunction and OHRQoL (Bakke et al. 2007; Thomson et al. 2013). Being structured into various domains and parts, allows the investigation of each element of orofacial dysfunction and OHRQoL separately (Bakke et al. 2007; Thomson et al. 2013; Al-Riyami et al. 2016).

The presence or absence of oral habit(s), as reported by the child’s parent in the administrative chart, was confirmed by the parent’s answers for the habit domain of the interview part of NOT-S items for the sucking habit, nail biting, and bruxism habits, and by the nose breathing domain of examination part of NOT-S for the mouth breathing habit.

In the present study, the children in the habit practicing group recorded a median of three (range 1–5), which was significantly higher (*p* = 0.00) than the habit free group (median = 0.05, range 0–2) in the total NOT-S, a result that goes in accordance with Leme et al. (2013) who conducted their research on Brazilian children aged from eight to 14 years, and found that the habit group scored a median of three (IQR: 2), while the habit free group scored a median of two (IQR: 2) total NOT-S, with a statistically significant difference between the two groups (*p* < 0.001).

Parental–Caregiver Perception Questionnaire recorded a median of six (range 1–16) in the habit practicing group, while the habit free group recorded a median of four (range 1–8), with a statistically significant difference (*p* = 0.014), which nearly matched the result of (Leme et al. 2013) who used Child Perception Questionaire _8–10_ (CPQ _8–10_), as an assessment tool for OHRQoL, in which the habit group scored a median of 12 (IQR:13), and the habit free group scored a median of eight (IQR: 10).

Concerning the sensory domain of the interview part of NOT-S, the habit practicing group scored a higher mean of 0.47 (0.51), in comparison to the habit free group which scored a mean of 0.13 (0.35), (*p* = 0.005). The sensory domain inquires about two items: “gagging sensation while brushing the teeth” and “putting too much food in the mouth that becomes difficult to chew”. This result goes in consistence with Leme et al. (2013) who suggested that higher gagging reflex in the habit group could be explained by the association between practicing oral habits and anxiety (Ghanizadeh 2008; Leme et al. 2014; Silva et al. 2019), one form of which is dental anxiety, that could be manifested as gag reflex in response to any tactile stimulation, as in teeth brushing (Schroeder and Santibanex 1978; Bassi et al. 2004; Almoznino et al. 2016).

The chewing and swallowing domain of the interview part of NOT-S was recorded as “taking prolonged time eating the main meal” and “swallowing large bits without proper chewing”. The habit practicing group scored a mean of 0.5 (0.5) versus 0.07 (0.25) for the habit free group. This goes in agreement with the score of the functional limitation part of P-CPQ, which contained two items; “difficulty in chewing” and “consuming a long time in eating”, ensuring the consistency of the collected data. The habit practicing group scored a mean of 2.93 (2.2) in the functional limitation part, while the habit free group scored 1.53 (1.46) (*p* = 0.011). The compromised masticatory function in the habit practicing group children could be explained by the change in jaw and tongue kinetics due to neuroplastic change in the primary motor cortex which participates in the sensorimotor regulation of the masticatory and swallowing processes, resulting in an intraoral alternation of tongue posture and impaired sensorimotor function associated with the change in the freeway space (Mason 2005; Avivi-Arber et al. 2011; Avivi-Arber and Sessle 2018). Another explanation is the association of the malocclusion with practicing oral habits (Kolawole et al. 2019), that impaired the normal masticatory function and directly affects the child’s OHRQoL (Liu et al. 2009; Sardenberg et al. 2013).

Concerning the breathing domain of the interview part of NOT-S, represented by “snoring during sleep”, the mean score was found to be 0.13 (0.35) and 0.07 (0.25) in the habit practicing group and the habit free group, respectively. Snoring in the breathing domain was reported by four children in the habit practicing group, three of them were mouth breathers (60% of mouth breathers in the habit practicing group) and one was a nail biter. This finding goes in agreement with (Izu et al. 2010) who stated that 58% of mouth breathing children reported primary snoring during the night time. On the other hand, it goes in contrast with Leme et al. (2013) who found no difference in the breathing domain mean scores between the habit group and the habit free group. This contrast in findings between the current study and Leme et al. study could be due to the recruitment of the habit group sample in Leme et al. study based on the habits domain of NOT-S only, while in the current study the habit practicing group children were recruited based on both the habit and the nose breathing domains of NOT-S, in which the mouth breathers were included in the habit practicing group in this study, but not in Leme et al. study.

The drooling domain of the interview part of NOT-S means scores were 0.16 (0.38) and 0.03 (0.18) in the habit practicing group and the habit free group, respectively. The dryness of the mouth domain of the interview part of NOT-S means scores were 0.43 (0.5) for the habit practicing group, and 0.017 (0.38) for the habit free group. Drooling and dryness of the mouth, in the habit practicing group, could be due to the lack of the proper lip seal as a consequence of the increased freeway space beyond the physiologic level as a result of practicing oral habits (Mason 2005). Also, this finding could be explained by the presence of a foreign object in the mouth (finger, lip, or nail) which stimulates salivary flow leading to drooling, and the mouth being opened most of the time leading to dryness of the mouth.

Except for the nose breathing domain of the examination part of NOT-S, which inquires about mouth breathing, both study groups were almost the same in all other examination part domains, which is justified by the healthy sample. The presence of lisping at letters (R, S, TH) was found to be equal in the two groups, justified by the age group in which the shedding of the primary incisors occurs, causing the inability to properly pronounce those letters. The lip incompetence was observed in one child in the control group, due to maxillary protrusion, which could be related to skeletal malocclusion rather than due to practicing oral habits.

The oral symptom part of P-CPQ represents two items: “pain sensation” and “presence of food debris in the mouth”. The higher score of the oral habit practicing group in this part could be explained by the association of oral habits with malocclusion (Reyes Romagosa et al. 2014; Kolawole et al. 2019), which in consequence results in temporomandibular disorders (Michelotti et al. 2020) and food stagnation in the mouth. The strain on muscles and temporomandibular joints which are resulted from practicing oral habits could explain the pain in orofacial structure (Karibe et al. 2015).

Concerning the emotional well-being of P-CPQ, which inquires about two items, “being upset”, and “being irritable or frustrated”, the higher score reported in the habit practicing group, 0.47 (0.94), versus the habit free group, 0.23 (0.68), could be explained by the association between the psychological disturbance and practicing oral habits (Manfredini et al. 2004; Ghanizadeh 2008; Leme et al. 2014). This finding agrees with Leal et al. (2016)***,*** who concluded in their study that mouth breather children are found to have a low quality of life aspects, such as being sad or blue in their day-by-day life, and being teased by other children for the way they breathe.

The social well-being part of P-CPQ scored a comparable score in both study groups, with the habit practicing group scored a slightly higher mean value; this finding could be justified by the recruited sample age group, in which the children’s cognitive abilities are not yet well developed to interpret their social limitations which resulted from practicing oral habit.

A moderately positive correlation between total NOT-S and total P-CPQ scores in the habit practicing group (*R* = 0.384, *p* = 0.036), suggests an existing positive relationship between the two variables, as a result of practicing oral habits, a finding that goes in agreement with Leme et al. 2013, who reported a moderately positive correlation between NOT-S score and CPQ _8–10_ (*R* = 0.32, *p* < 0.001). The positive correlation between orofacial dysfunction and the child’s OHRQoL was previously reported by Montes et al. (2019) in children with unilateral cleft lip and palate, and Sardenberg et al. (2017) who concluded in their study that school children suffering from orofacial dysfunction, assessed by NOT-S, are having inferior OHRQoL. According to the reported findings of the present study, orofacial dysfunction is found to negatively influence the child’s OHRQoL, especially in terms of chewing and swallowing, and dryness of the mouth domains of the interview part of the NOT-S.

In the current study, the habit practice group was found to be 7.4 and 1.5 times the scores of the habit free group in developing orofacial dysfunction, and having inferior OHRQoL, respectively, confirming the association between practicing oral habits and both developing orofacial dysfunction, and having inferior OHRQoL.

The clinical relevance of this research could be demonstrated in terms of the importance of the proper interceptive measures to break the habit, in the appropriate age range, which will protect the child from the higher risk of developing orofacial dysfunction and prevent the inferior perception of the child towards his OHRQoL.

The current findings of this study are expected to be applicable to different populations, as children are expected to have a comparable orofacial growth pattern which would be affected in the same manner by practicing oral habits, and further studies on other populations are recommended to test this assumption.

Among the limitations of the current study are the relatively low sample size and lack of baseline clinical assessment of the habit in children in terms of intensity, frequency of habit practicing per day, muscle activity or passivity during practicing the habit, habitual or obstructive mouth breathing, and the correlation of this clinical picture with the severity of orofacial dysfunction.

## Conclusion

Children practicing one or more of the deleterious oral habits suffer from a worse orofacial dysfunction condition and show inferior OHRQoL, in comparison to their counterparts, who do not practise any of the deleterious oral habits.

An existing association between the degree of orofacial dysfunction and OHRQoL in children practicing oral habit(s) is suggested.

## Data Availability

Raw data (master table) is available upon request.
